# Collagen‐derived peptides modulate CD4^+^ T‐cell differentiation and suppress allergic responses in mice

**DOI:** 10.1002/iid3.213

**Published:** 2018-02-01

**Authors:** Akihiko Nishikimi, Yoh‐ichi Koyama, Sayaka Ishihara, Shusaku Kobayashi, Chisa Tometsuka, Masashi Kusubata, Kumiko Kuwaba, Osamu Hayashida, Shunji Hattori, Koko Katagiri

**Affiliations:** ^1^ Department of Biosciences School of Science Kitasato University Sagamihara Kanagawa Japan; ^2^ Research Institute of Biomatrix Nippi Inc. Toride Ibaraki Japan; ^3^ Institute for Animal Reproduction Kasumigaura Ibaraki Japan

**Keywords:** Active cutaneous anaphylaxis model, collagen peptide, T cells

## Abstract

**Introduction:**

Collagen peptides have been widely used as a food supplement. After ingestion of collagen peptides, oligopeptides containing hydroxyproline (Hyp), which are known to have some physiological activities, are detected in peripheral blood. However, the effects of collagen‐peptide administration on immune response are unclear. In the present study, we tested the effects of collagen‐peptide ingestion on allergic response and the effects of collagen‐derived oligopeptides on CD4^+^ T‐cell differentiation.

**Methods:**

BALB/c mice fed a collagen‐peptide diet were immunized with ovalbumin (OVA), and their serum IgE and IgG levels, active cutaneous anaphylaxis, and cytokine secretion by splenocytes were examined. Naive CD4^+^ T cells were stimulated with anti‐CD3 and anti‐CD28 in the presence of collagen‐derived oligopeptides, and the expression of IFN‐γ, IL‐4, and Foxp3 was analyzed.

**Results:**

In an active anaphylaxis model, oral administration of collagen peptides suppressed serum OVA‐specific immunoglobulin E (IgE) production and diminished anaphylaxis responses. In this model, the ingestion of collagen peptides skewed the pattern of cytokine production by splenocytes toward T‐helper (Th) type 1 and regulatory T (Treg) cells. In vitro T‐helper cell differentiation assays showed that Hyp‐containing oligopeptides promoted Th1 differentiation by upregulating IFN‐γ‐induced signal transducer and activator of transcription 1 (STAT1) signaling. These oligopeptides also promoted the development of Foxp3^+^ Treg cells in response to antigen stimulation in the presence of TGF‐β.

**Conclusions:**

Collagen‐peptide ingestion suppresses allergic responses by skewing the balance of CD4^+^ T cells toward Th1 and Treg cells and seems to be a promising agent for preventing allergies and inflammatory diseases.

## Introduction

In allergic diseases, the balance among the subsets of effector CD4^+^ T cells and regulatory T (Treg) cells plays a crucial role in controlling pathological conditions. Inappropriate expansion of T‐helper (Th) type 2 cells in response to allergens results in atopic sensitization and allergic diseases 1, 2, 3. Th2‐associated cytokines, including interleukin (IL)‐4, ‐5, and ‐15, promote the production of IgE to allergens and other chemical mediators, which are responsible for subsequent hypersensitivity reactions. The polarization of naive CD4^+^ T cells into functionally distinct subsets upon antigen stimulation depends on many parameters, including the status of cells and cytokines in the microenvironment 4, 5. Development of each subset of CD4^+^ T cells is mutually exclusive, and IFN‐γ, a Th1‐associated cytokine, is thought to inhibit the development of Th2 pathologies in Th1 dominance 6. Recent studies have shown that Treg cells contribute to maintaining healthy immune responses to allergens by suppressing several immune responses, including proliferation of antigen‐specific T‐helper cells and innate effector cells, and by producing Th1‐ and Th2‐associated cytokines 7, 8.

Collagen is one of the major constituents of the extracellular matrix. Heat‐denatured collagen, referred to as gelatin, is widely used in foods and pharmaceuticals. Collagen peptides, prepared by partially hydrolyzing gelatin, have been used as a food supplement to improve joint disorders and skin conditions 9, 10. Collagen and its derivatives specifically contain hydroxyproline (Hyp), an imino acid formed by post‐translational hydroxylation of proline residues. Peptides containing Hyp are relatively resistant to digestion by digestive enzymes and protein‐degrading enzymes in the circulatory system; therefore, collagen‐derived oligopeptides remain in circulation after ingestion of collagen peptides 11. Among these peptides, prolylhydroxyproline (Pro‐Hyp) is a major constituent of collagen‐derived peptides and has been shown to have some physiological activities 12, 13. For example, it enhances the growth and hyaluronic‐acid production of skin fibroblasts 14, 15, promotes differentiation of osteoblastic cells 16, and suppresses mineralization in chondrocytes 17. Hydroxyprolylglycine (Hyp‐Gly) is also found in human blood at relatively high concentrations after oral administration of collagen peptides and has been shown to stimulate the growth of skin fibroblasts 18 and promote myoblast differentiation and myotube hypertrophy 19.

The involvement of collagen‐derived peptides in inflammatory disease is suggested by the observation that both endogenously­ generated and orally administrated Pro‐Hyp are deposited at local inflammatory sites in a murine contact‐dermatitis model 20. Collagen‐derived oligopeptides, including Pro‐Hyp, have also been shown to exert chemotactic activities on fibroblasts 21, neutrophils 22, and monocytes 23, all of which play important roles in inflammation and wound healing. Indeed, supplemental ingestion of collagen peptides has been shown to suppress ultraviolet‐induced skin damage and erythema in mice and humans 24, 25 and to promote wound healing in rats and humans 26, 27, 28. It has also been demonstrated that ingestion of collagen peptides improves human immunological status as assessed by 14 immunological parameters, suggesting that collagen peptides or their derivatives modulate immune responses and functions 29.

The present study examined the effects of collagen‐peptide ingestion on immune and allergic responses and found that oral administration of collagen peptides reduced the production of antigen‐specific IgE and anaphylaxis responses in a murine anaphylaxis model. This study also demonstrated that Pro‐Hyp and Hyp‐Gly promoted the development of CD4^+^ T cells toward Th1 and Treg cells in vitro, which is thought to contribute to reducing immune responses mediated by Th2.

## Materials and Methods

### Chemicals and reagents

Pro‐Hyp and Hyp‐Gly were obtained from Bachem (Bubendorf, Switzerland). IFN‐γ, IL‐4, and TGF‐β were purchased from R&D Systems (Minneapolis, MN, USA). Ovalbumin (OVA) was purchased from Sigma‐Aldrich (St. Louis, MO, USA).

### Animal experiments

BALB/c and C57BL/6 mice were purchased from Japan SLC (Hamamatsu, Japan) and maintained under specific pathogen‐free conditions in the animal facility of Kitasato University. The mice were housed in individual cages under a 12‐h/12‐h light‐dark cycle at 23 ± 5°C throughout the experimental period. The mice were divided into two groups (control and collagen) and fed a diet based on AIN‐93M (powdered form; Oriental Yeast; Tokyo, Japan), which contained only casein as a protein source. The control feed contained 18% casein, and the collagen‐peptide feed contained 14% casein and 3.08% collagen peptides, substituting on an equal nitrogen basis. To equalize protein administration among the animals, food intake was moderately restricted to approximately 90% of free‐feeding weight. Mice (age 4 weeks) were fed 3.0 g/day on days 1–3, 3.5 g/day on days 4–7, 4.0 g/day on days 8–17, and 4.5 g/day after day 18. Water was provided ad libitum. Mice with free access to a standard diet and water were used in the experiments of in vitro T‐cell differentiation and signal transduction. All animal protocols were approved by the Animal Care and Use Committee of Kitasato University (No. 1711).

### OVA‐induced allergy model

BALB/c mice were injected intraperitoneally with 10 μg of OVA in 100 μL of PBS mixed with 200 μL of Imject‐Alum (Thermo Fisher Scientific; Waltham, MA, USA) on days 7, 14, and 21 of either control or collagen feeding. Serum was collected from the tails 5 days after the last immunization, and total and OVA‐specific immunoglobulin were measured using enzyme‐linked immunosorbent assay (ELISA). Active cutaneous anaphylaxis was induced as described previously 30. Briefly, 7 days after the last injection, mice were challenged by intradermal injection of 30 μg of OVA in PBS to the right ear and PBS alone to the left ear. Ear thickness was measured with an engineer's micrometer (Ozaki MFG; Tokyo, Japan) 30 min after this challenge.

### ELISA for antibodies

Levels of total immunoglobulin E (IgE) and immunoglobulin G (IgG) were measured with a mouse IgE and IgG ELISA quantitation set (Bethyl Laboratories; Montgomery, TX, USA), respectively, according to the manufacturer's instructions. To measure OVA‐specific IgG levels, the wells of Nunc MaxiSorp immunoplates (Thermo Fisher Scientific) were coated with 5 μg/mL of OVA in 50 mM of carbonate buffer (pH 9.5). After blocking with Tris‐buffered saline (TBS; 20 mM of Tris‐HCl, 150 mM of NaCl, pH 7.5) containing 5% skim milk, test samples diluted in TBST (TBS supplemented with 0.05% Tween‐20) were added. Bound IgG was detected with peroxidase‐conjugated anti‐mouse IgG antibody (Southern Biotech; Birmingham, AL, USA) and 3,3′,5,5′‐tetramethylbenzidine (TMB) substrate (Vector Laboratory; Burlingame, CA, USA). To measure OVA‐specific IgE levels, the wells were coated with 200 μg of OVA. After blocking and sample treatment, bound IgE was detected with biotinylated anti‐mouse IgE and peroxidase‐conjugated streptavidin. The levels of OVA‐specific IgG and IgE were estimated by comparison with serum from hyper‐immunized mice, namely BALB/c mice immunized with three doses of OVA mixed with Imject‐Alum. The OVA‐specific IgG and IgE levels of this hyper‐immunized serum were estimated as 5 × 10^7^ and 1 × 10^3^ units/mL, respectively.

### Cell cultures for cytokine production

BALB/c mice were immunized with 10 μg of OVA mixed with Imject‐Alum on day 7 of either control or collagen feeding. Splenocytes were isolated from mice 10 days after immunization, cultured in medium (RPMI1640 with 10% FBS, 0.05 mM of 2‐mercaptoethanol, penicillin, and streptomycin) supplemented with 10 μg/mL of OVA at 2.5 × 10^6^ cells/mL, and cultured in a 24‐well plate (1 mL/well) for 3 days. Levels of cytokines in the supernatant were determined using a mouse Th1/Th2/Th17 cytokine kit (BD Biosciences; San Diego, CA, USA) and Mouse IL‐13 Flex Set (BD Biosciences).

### In vitro differentiation of CD4^+^ T cells

CD4^+^ T cells were isolated from the spleen and peripheral lymph nodes with a CD4^+^ T‐cell isolation kit (Miltenyi Biotec; Bergisch Gladbach, Germany) according to the manufacturer's instructions. To differentiate T‐helper cells, isolated cells were suspended in medium and cultured in 24‐well plates coated with anti‐CD3 (2c11; 1 μg/mL)/anti‐CD28 (37.51; 1 μg/mL) (1 × 10^6^ cells/well) in the presence or absence of 200 μM of peptides. This experiment used dialyzed FBS (Thermo Fisher Scientific) to avoid the effects of small peptides contained in the FBS. On day 4, differentiated cells were re‐stimulated with plate‐bound anti‐CD3 for 8 h in the presence of 10 μg/mL of brefeldin A (Sigma–Aldrich) during the final 2 h of the culture. Re‐stimulated cells were fixed in 4% paraformaldehyde, permeabilized in PBS containing 0.5% saponin, stained with fluorescein isothiocyanate (FITC)‐conjugated anti‐IFN‐γ (XMG1.2) and phycoerythrin (PE)‐conjugated anti‐IL‐4 (11B11), and analyzed on a flow cytometer (Gallios; Beckman Coulter; Brea, CA, USA). To induce Treg cells, CD4^+^ T cells (1 × 10^6^ cells/well) were cultured with or without 200 μM of peptides or free amino acids in 24‐well plates coated with anti‐CD3 and anti‐CD28 in the presence of TGF‐β1 (1 ng/mL) and anti‐IFN‐γ (1 μg/mL) for 5 dayd. Cells were stained for intracellular Foxp3 using the anti‐mouse/rat Foxp3 staining set APC (Thermo Fisher Scientific) and analyzed using flow cytometry.

### Reverse transcription (RT)‐PCR

Total RNA was extracted from naive CD4^+^ T cells using Trizol reagent (Thermo Fisher Scientific) and then treated with DNase I. RNA (1 μg) was reversely transcribed using the PrimeScript RT reagent Kit (Takara Bio; Shiga, Japan). Fetal mouse cDNA (day 10.5) was purchased from Wako Pure Chemical (Osaka, Japan). Each cDNA was amplified by polymerase chain reaction (PCR) using rTaq polymerase (Toyobo; Osaka, Japan) at 94°C for 1 min, followed by 35 cycles at 94°C for 30 sec, 60°C for 30 sec, and 72°C for 1 min. The PCR primer pairs were as follows: *pept1* (forward primer 5′‐tcacagaccacgaccacaat‐3′ and reverse primer 5‐ccccgttgatagccaaataa‐3′); *pept2* (forward primer 5′‐atcctgcagtgcattgtgaa‐3′ and reverse primer 5′‐ctgctgctgtaaccaggaca‐3′); *pht1* (forward primer 5′‐ccgtgttcttggctctgatt‐3′ and reverse primer 5′‐ccaccagcttgtccttcagt‐3′); and *Ci1* (forward primer 5′‐gttgcggtgatcctgattct‐3′ and reverse primer 5′‐agctgaggcactgtctggtt‐3′).

### Immunoblot analysis

Analysis of the activation of signal transducer and activator of transcription (STAT) 1 and STAT6 was performed as previously described 31, with slight modifications. Briefly, CD4^+^ T cells from BALB/c mice (for STAT1 activation) or C57BL/6 (for STAT6 activation) were stimulated with plate‐bound anti‐CD3 (0.5 μg/mL) in the presence of anti‐IFN‐γ (1 μg/mL, for STAT1 activation) or anti‐IL‐4 (1 μg/mL, for STAT6 activation) for 2 h. The cells were washed with PBS, suspended in RPMI1640 medium, and stimulated with recombinant IFN‐γ (250 U/mL) or IL‐4 (1 U/mL). Pro‐Hyp peptide or free amino acids indicated in the figure were added at a concentration of 200 μM throughout the course of the experiment. Cell lysates were subjected to immunoblotting with anti‐phospho STAT1 (Cell Signaling Technology; Beverly, MA, USA), anti‐STAT1 (Cell Signaling Technology), anti‐phospho STAT6 (Cell Signaling Technology), and anti‐STAT6 (BD Biosciences).

### In vitro suppression assay

CD25^+^ cells were magnetically sorted with MACS system from BALB/c CD4^+^ T cells cultured in the Treg condition in the presence or absence of 200 μM Pro‐Hyp and used as Treg cells. CD4^+^ T cells isolated from spleen and peripheral lymph nodes of BALB/c mice were labeled with 1 μM carboxyfluorescein diacetate succinimidyl ester (CFSE). Labeled CD4^+^ T cells (5 × 10^6^ cells) were cultured with or without Treg cells in 96‐well round bottom plate with anti‐CD3 (1 μg/mL) and anti‐CD28 (1 μg/mL). After 48 h, proliferation of CD4^+^ T cells was analyzed by FACS for dilution of CFSE.

Statistical analysisDifferences between two groups were analyzed using a one‐tailed Student's *t*‐test. Comparisons among more than two groups were performed using a Kruskal–Wallis test followed by a Dunn's multiple‐comparison test.

## Results

### Oral administration of collagen peptides reduced antigen‐specific IgE production and anaphylaxis reactions

**Figure 1:**
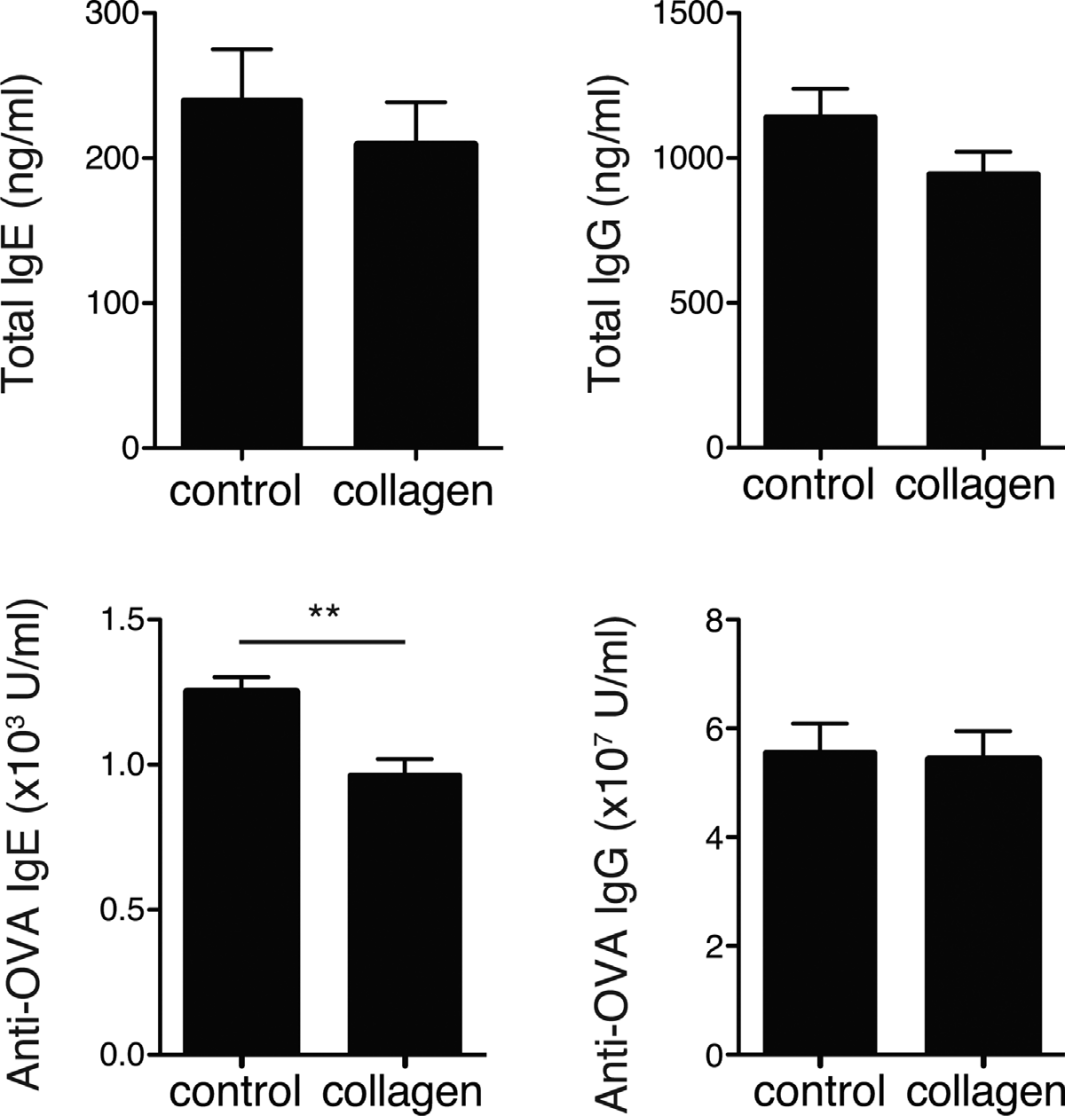
Effects of collagen‐peptide administration on humoral immune responses. BALB/c mice fed a diet with or without collagen peptides were intraperitoneally injected with 10 μg of OVA and Alum at weeks 1, 2, and 3. Serum was collected from each mouse, and total and OVA‐specific IgE and IgG were measured using ELISA. Values are means ± SEM (*n* = 16) obtained by two independent experiments. **P < 0.01.

During the experimental period, mice kept on the feeding procedure described above showed normal growth. No differences in growth rate and water intake were found between the two groups of animals (Fig. S1). After 1, 2, and 3 weeks of casein or collagen feeding, the mice were intraperitoneally injected with OVA in Alum. Serum was collected 5 days after the last immunization, and total and OVA‐specific IgE and IgG were measured. The OVA‐specific IgE level in the collagen group was significantly lower than that in the control group, although the total IgE level was comparable between the two groups (Fig. 1). Both the total and OVA‐specific IgG levels were unaffected by collagen‐peptide feeding.

**Figure 2:**
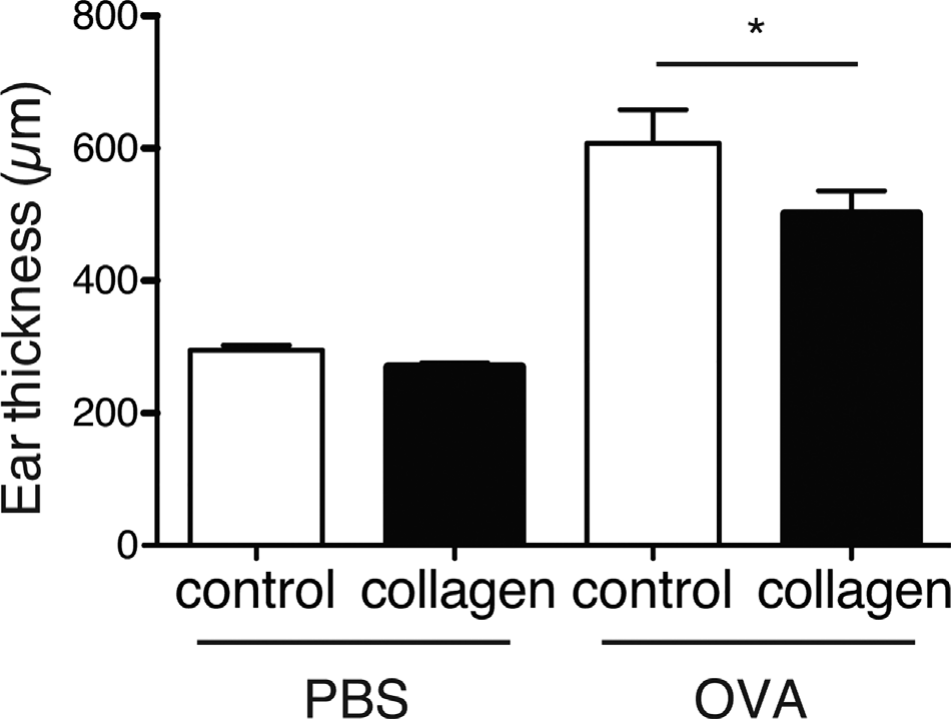
Suppression of anaphylaxis by collagen‐peptide administration. BALB/c mice were fed and immunized as described in Figure 1. Mice were subcutaneously challenged using OVA dissolved in PBS (right ear) and PBS alone (left ear) 7 days after the last immunization. Ear thickness was measured 30 min after challenge. Values are means ± SEM (*n* = 8). Representative data of two independent experiments were shown. *P < 0.05.

The reduction in production of antigen‐specific IgE in the collagen group led us to examine whether collagen‐peptide administration can suppress allergic reactions in the active cutaneous anaphylaxis model. Mice were immunized as described above and challenged subcutaneously in the ear with OVA 7 days after the last immunization. Collagen‐peptide administration significantly reduced ear swelling, indicating that collagen peptides have an inhibitory effect on allergic reactions (Fig. 2).

### Oral feeding of collagen peptides favors Th1 cytokine secretion

**Figure 3:**
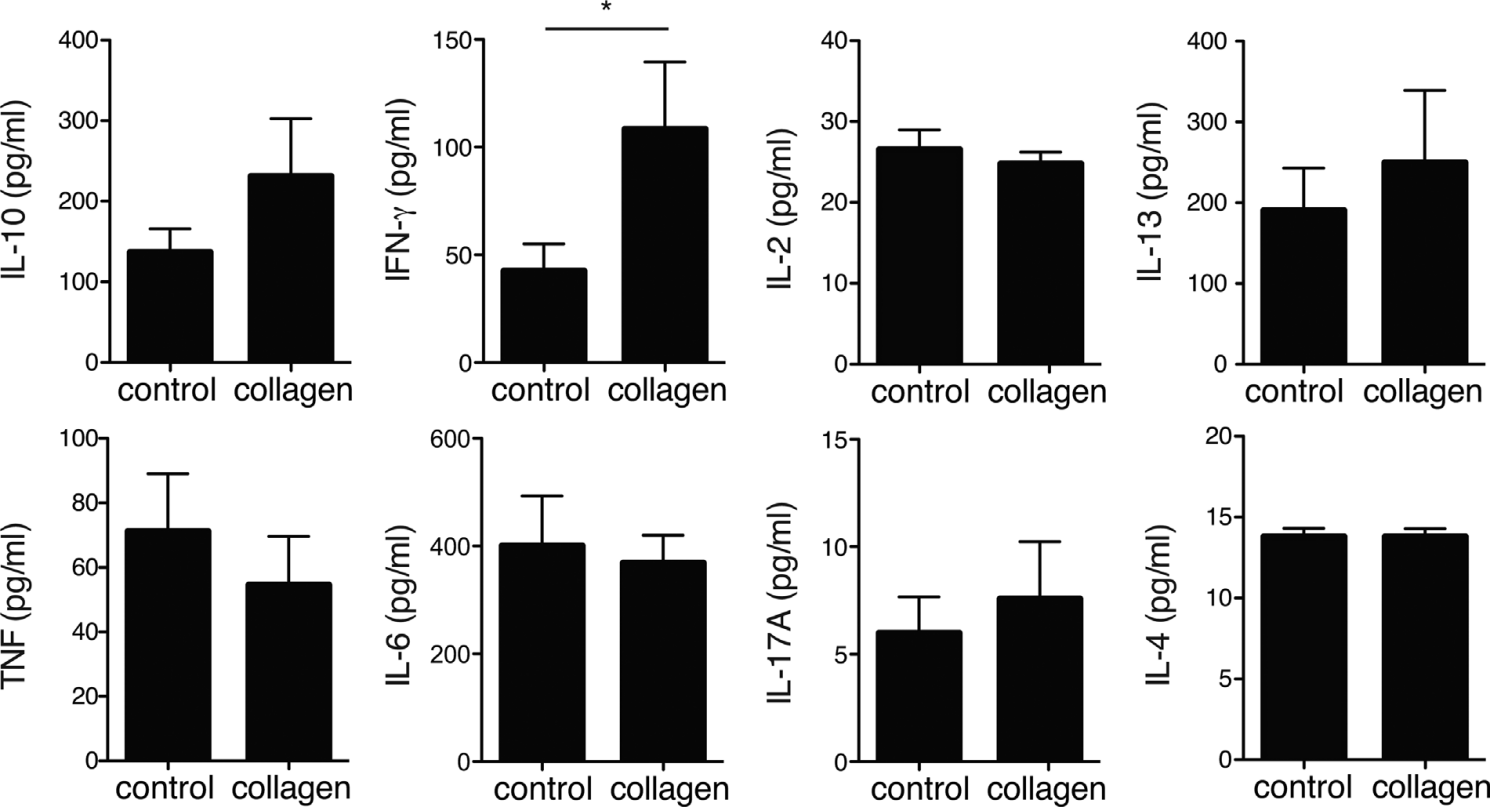
Effects of collagen‐peptide administration on cytokine production by splenocytes. BALB/c mice fed a diet with or without collagen peptides were intraperitoneally injected with 10 μg of OVA and Alum. Splenocytes were collected 10 days after the injection and cultured with 10 μg/ml of OVA for 72 h before cytokine levels in culture supernatants were determined. Values are means ± SEM (*n* = 6). Representative data of two independent experiments were shown. *P < 0.05.

To examine whether the administration of collagen peptides modulates immune responses, we collected splenocytes from control or collagen‐fed mice 10 days after the primary immunization, cultured the cells in the presence of OVA, and measured the cytokine levels in the supernatant. As Figure 3 shows, collagen‐peptide feeding significantly enhanced IFN‐γ secretion by splenocytes compared to the amount secreted by the control group (Fig. 3). Secretion of IL‐10 was also enhanced in the collagen group but to a degree that was not statistically significant. In contrast, ingestion of collagen peptide did not affect the secretion of type 2 cytokine, such as IL‐13. Production of IL‐4 and IL‐17A was not sufficient to enable analysis of the difference between the two groups. Collagen‐peptide feeding did not significantly affect secretion of IL‐2, IL‐6, or TNF. These data suggested that collagen peptides have the potential to modulate the Th1/Th2 balance toward a Th1‐dominant state, because IFN‐γ is one of the key cytokines inducing Th1‐dominant immunity.

### Collagen‐derived dipeptides skew CD4^+^ T‐cell development toward Th1 by enhancing STAT1 signaling

**Figure 4:**
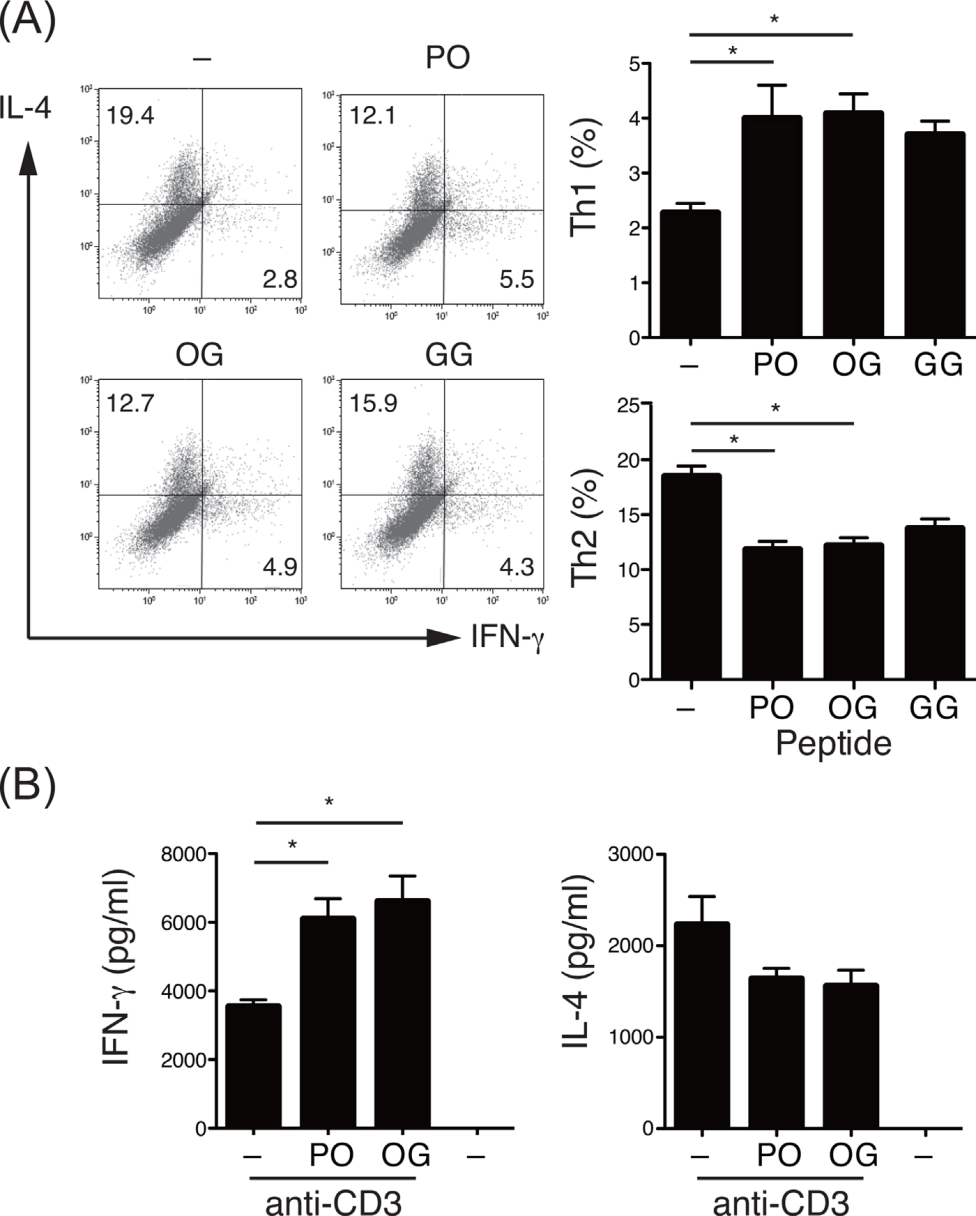
Effects of collagen‐derived dipeptides on the differentiation of T‐helper cells. (A) Production of IFN‐γ and IL‐4 by BALB/c CD4^+^ T cells stimulated with plate‐bound anti‐CD3 and anti‐CD28 in the presence or absence of 200 μM of Pro‐Hyp (PO), Hyp‐Gly (GO), or Gly‐Gly (GG). Cells were re‐stimulated with plate‐bound anti‐CD3 on day 4 and subjected to intracellular cytokine staining followed by flow cytometry. Numbers in quadrants indicate percent of cells in each. The graph shows the percentage of CD4^+^ T cells differentiated into Th1 and Th2 (*n* = 5). (B) BALB/c CD4^+^ T cells stimulated with plate‐bound anti‐CD3 and anti‐CD28 in the presence or absence of 200 μM of indicated peptides for 3 days, and then cytokine levels in culture supernatants were determined (*n* = 3). Values are means ± SEM. Representative data of at least two independent experiments were shown. *P < 0.05.

After ingesting collagen peptides, a considerable amount of Hyp is absorbed and accumulated in the blood as various forms of oligopeptides, including Pro‐Hyp and Hyp‐Gly, which are known to have several physiological activities. It has been thought that these oligopeptides interact with their effector molecules after incorporation into cells through proton‐coupled active transporters, including peptide transporter (PEPT) 1, PEPT2, peptide/histidine transporter (PHT) 1, and Ci1 (PHT2 in humans) 32. RT‐PCR analysis showed that *Pept2*, *Pht1*, and *Ci1* were expressed in naive CD4^+^ T cells (Fig. S2), suggesting their ability to use oligopeptides. To examine the effects of these collagen oligopeptides on the development of T‐helper cells, we stimulated CD4^+^ T cells with anti‐CD3 and anti‐CD28 in the presence of Pro‐Hyp or Hyp‐Gly peptides and measured the expression of IL‐4 and IFN‐γ. As expected from the in vivo experiments, significantly higher frequencies of Th1 cells (IFN‐γ^+^) and lower frequencies of Th2 cells (IL‐4^+^) were detected in CD4^+^ T cells stimulated in the presence of Pro‐Hyp or Hyp‐Gly peptides compared to CD4^+^ T cells stimulated in the absence of peptides (Fig. 4A). The control peptide glycylglycine (Gly‐Gly) also slightly modulated differentiation into Th1 or Th2 cells compared to the no‐peptide group, but to a degree that was not statistically significant. In agreement with these results, CD4^+^ T cells stimulated with anti‐CD3 and anti‐CD28 in the presence of Pro‐Hyp or Hyp‐Gly have been shown to produce significantly higher levels of IFN‐γ (Fig. 4B). Production of IL‐4 was reduced in the presence of Pro‐Hyp or Hyp‐Gly, although the difference was not statistically significant. These results indicated that the presence of collagen‐derived dipeptides skews Th1/Th2 differentiation toward Th1.

**Figure 5:**
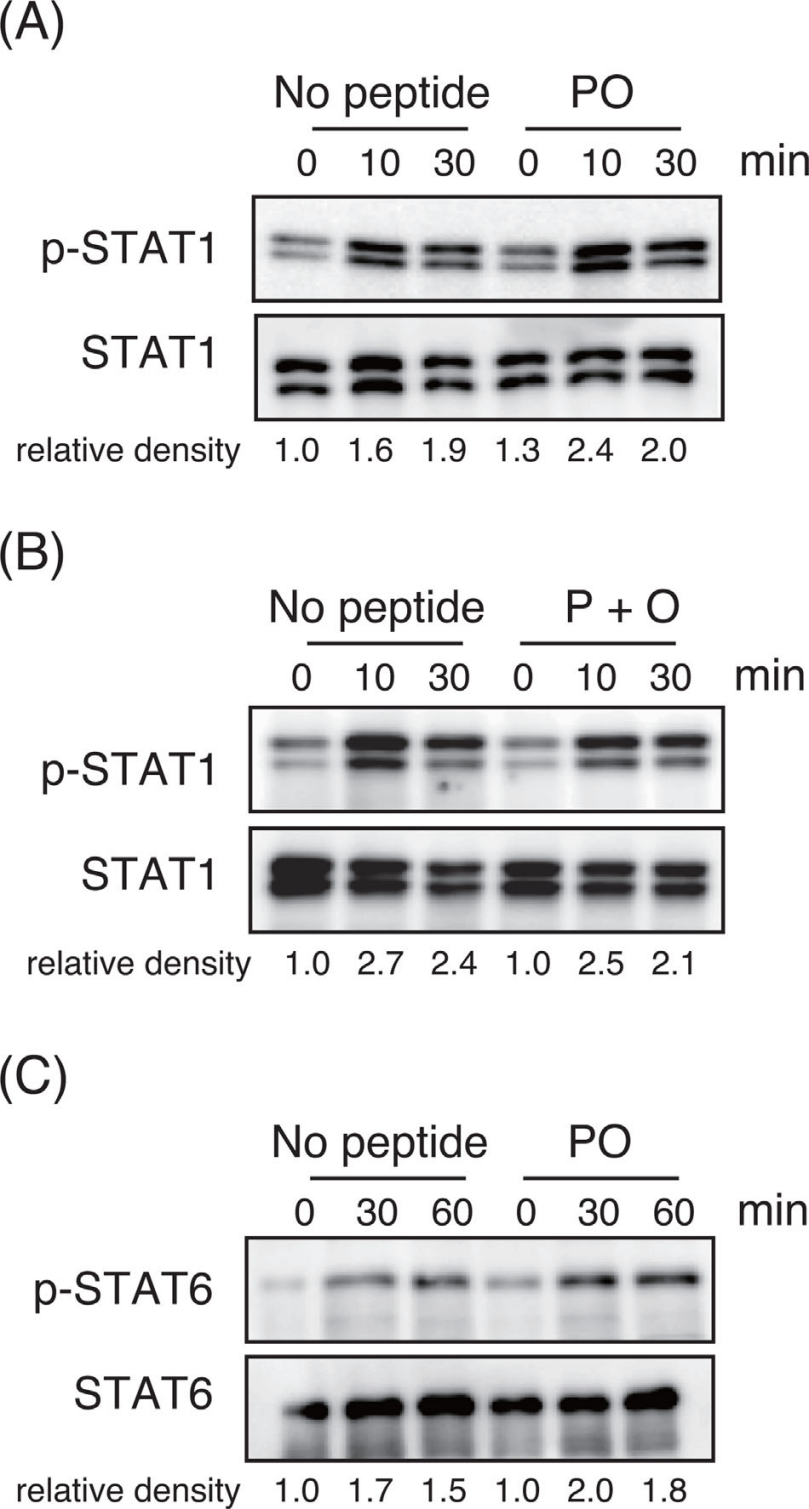
Effects of collagen‐derived dipeptides on cytokine‐induced STAT signaling. (A and B) BALB/c CD4^+^ T cells pre‐activated for 2 h with immobilized anti‐CD3 in the presence or absence of 200 μM of (A) Pro‐Hyp (PO) or (B) free Pro and Hyp (P + O) were stimulated with IFN‐γ, and phosphorylation of STAT1 was analyzed using immunoblotting. (C) C57BL/6 CD4^+^ T cells pre‐activated with immobilized anti‐CD3 in the presence or absence of 200 μM Pro‐Hyp were stimulated with IL‐4, and phosphorylation of STAT6 was analyzed using immunoblotting. The density value of each band was expressed as the phosphorylated versus total protein after normalization of the 0‐min value of “no peptide” to an arbitrary unit of 1. Similar results were obtained at least two independent experiments.

To explore the mechanism by which collagen‐derived dipeptides enhance the development of Th1 cells, we analyzed the effects of these peptides on the signaling events induced by IFN‐γ. We pre‐activated CD4^+^ T cells with immobilized anti‐CD3 in the presence or absence of the Pro‐Hyp peptide for 4 h and then stimulated them with IFN‐γ. The Pro‐Hyp treatment slightly but clearly enhanced the STAT1 phosphorylation at 10 min after stimulation of IFN‐γ (Fig. 5A). However, the addition of free Pro and Hyp did not show such an effect (Fig. 5B). We detected no clear difference in STAT6 phosphorylation induced by IL‐4 in the presence or absence of Pro‐Hyp peptide when CD4^+^ T cells from Th1‐prone C57BL/6 mice were analyzed under similar conditions (Fig. 5C). These results suggest that Pro‐Hyp incorporated into CD4^+^ T cells enhances the events of STAT1 signaling, resulting in the promotion of Th1 differentiation.

### Collagen‐derived dipeptides promote development of Treg cells

**Figure 6:**
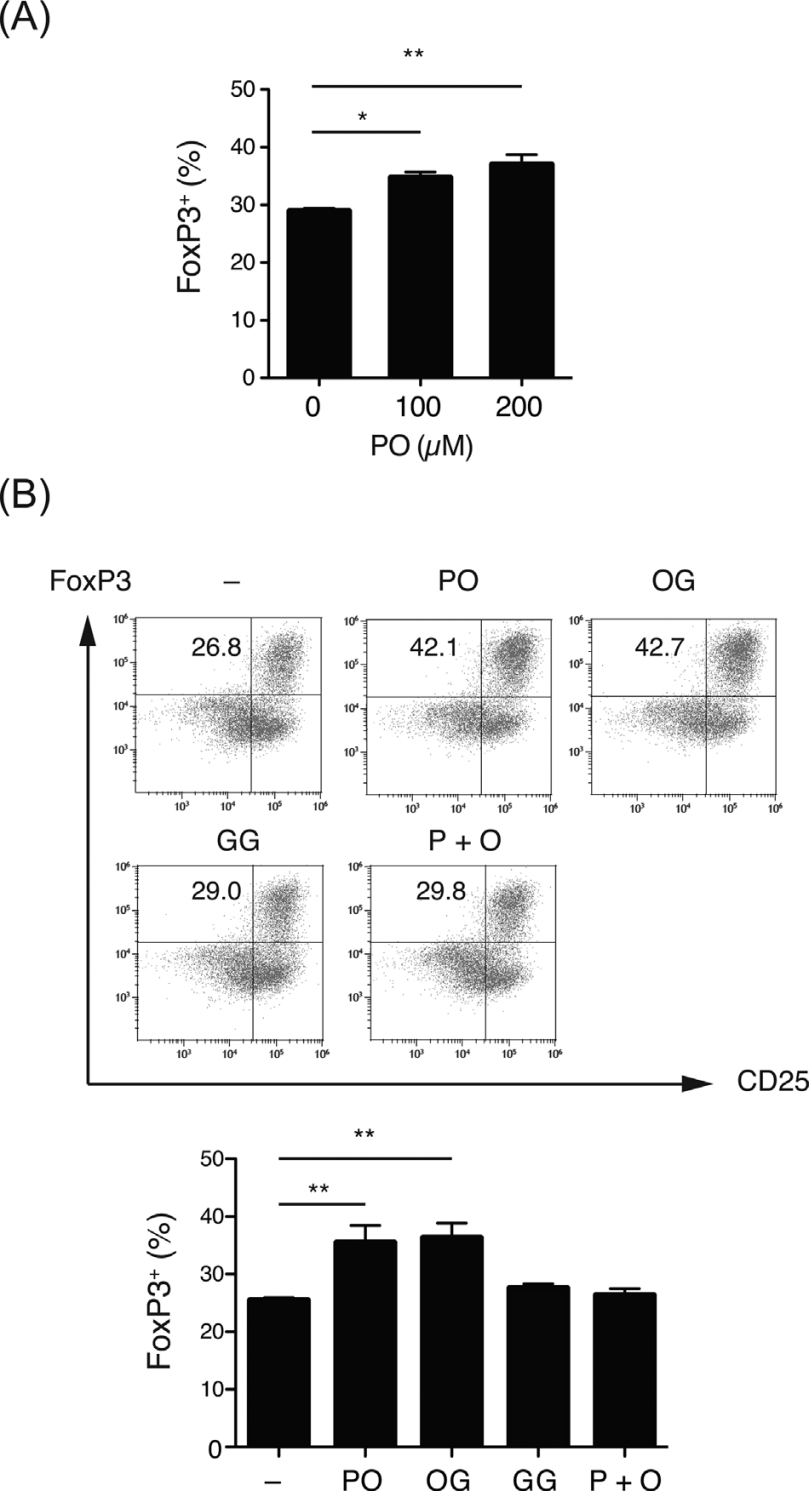
Effects of collagen‐derived dipeptides on the differentiation of Treg cells. (A) Naive BALB/c CD4^+^ T cells were stimulated with plate‐bound anti‐CD3/CD28 and TGF‐β under the indicated concentrations of Pro‐Hyp (PO), and the percentage of CD4^+^Foxp3^+^CD25^+^ Treg cells was analyzed using flow cytometry (*n* = 3). (B) Naive CD4^+^ T cells were stimulated as in (a) in the presence of 200 μM of Pro‐Hyp (PO), Hyp‐Gly (OG), Gly‐Gly (GG), and free Pro and Hyp (P + O) and analyzed using flow cytometry. Numbers in plots indicate the percentage of Foxp3^+^/CD25^+^ cells. The graph shows the percentage of CD4^+^Foxp3^+^CD25^+^ Treg cells (*n* = 4). Values are means ± SEM. Representative data of at least two independent experiments were shown. *P < 0.05, **P < 0.01.

Although the difference was not significant, ingesting collagen peptides promoted production of antigen‐induced IL‐10 by splenocytes (Fig. 3). IL‐10 has potential immunosuppressive capacity and is known to be produced by Treg cells. We examined whether collagen‐derived dipeptides promote the generation of Treg cells. Pro‐Hyp promoted the differentiation of Foxp3^+^ Treg cells when naive CD4^+^ T cells were stimulated using anti‐CD3, anti‐CD28, and TGF‐β. The effect was dose‐dependent, reaching maximal levels at 200 μM (Fig. 6A). A similar effect was observed with Hyp‐Gly, while the development of Treg cells was not affected by the addition of Gly‐Gly or free Pro and Hyp (Fig. 6B). CD4^+^CD25^+^ cells differentiated in the presence of Pro‐Hyp had potent suppressive function in vitro and produced equivalent levels of IL‐10 in response to anti‐CD3 and anti‐CD28 stimulation compared to those differentiated without dipeptide (Fig. S3). These results indicated that the uptake of Hyp‐containing dipeptides promoted antigen‐induced Treg development.

## Discussion

The present study demonstrated that intake of collagen peptides reduced allergic reactions mediated by IgE in an OVA‐induced active anaphylaxis model. This may be partly due to enhancement of STAT1 signaling in CD4^+^ T cells that incorporated collagen‐derived oligopeptides, including Pro‐Hyp and Hyp‐Gly, which resulted in predominant Th1‐type cytokine secretion. The Hyp‐containing peptides also promoted antigen‐induced generation of Treg cells. These results indicated that collagen peptides or their derivatives have the potential to skew antigen‐primed CD4^+^ T cells toward Th1 and Treg, which suppress the development of Th2 cells and Th2‐mediated allergic responses.

Recent evidence has emphasized the importance of Treg cells in maintaining immune homeostasis, including Th2 responses 7, 8. We have shown that treating naive CD4^+^ T cells with Pro‐Hyp or Hyp‐Gly under Treg‐polarizing conditions enhanced the development of Foxp3^+^ Treg cells. Naive CD4^+^ T cells are converted into Treg cells primarily in gut‐associated lymphoid tissues 33. These peripherally induced Treg cells play central roles in preventing inflammatory and allergic responses on mucosal surfaces. The immunosuppressive activities of intestinal Foxp3^+^ Treg cells are due at least in part to the secretion of IL‐10 34, 35, which suppresses production of antigen‐specific IgE 36 and inhibits mast cell and eosinophil activities 37, 38. In line with these findings, antigen‐induced IL‐10 production in splenocytes tends to be promoted by administration of collagen peptides. Interestingly, orally administrated Pro‐Hyp has been detected at the highest levels in the wall of the small intestine 39. Taken together, these results indicate that orally administrated collagen peptides may contribute to maintaining intestinal homeostasis and preventing allergic responses by promoting the induction of Treg cells in the gut.

Although the pathogenesis of allergic diseases cannot be explained simply by the Th1/Th2 paradigm, several lines of evidence have demonstrated that the Th1‐mediated immune response suppresses Th2 polarization and IgE production 40. IFN‐γ suppresses not only Th2 development but also Th2‐mediated effector functions that promote disease 41. Indeed, IFN‐γ and IL‐10 have been suggested as protecting allergic polysensitization in children 42. Thus, the reduction of allergic responses by collagen‐peptide ingestion observed in the present study may be partly due to skewing the Th1/Th2 balance in favor of Th1 and enhancing IFN‐γ production. Whole‐body radiography of rats has shown that orally administrated radiolabeled Hyp‐containing peptides (Pro‐Hyp and Gly‐Pro‐Hyp) accumulate in several organs, including the spleen, within 30 min of ingestion and are present at detectable levels for at least 24 h 39, 43. Since naive T cells are primed and develop into T‐helper cells in the spleen and other lymphoid organs, it is possible that the fate of CD4^+^ T cells is affected in collagen‐fed individuals, resulting in the promotion of differentiation into Th1 cells and IFN‐γ production.

It has been reported that collagen‐derived oligopeptides are transported into cells through peptide transporters, including PEPT and PHT proteins, and interact with effector proteins to modulate cellular signaling 19, 32, 44. The present study showed that Pro‐Hyp and Hyp‐Gly enhance IFN‐γ‐mediated STAT1 phosphorylation in pre‐activated CD4^+^ T cells, suggesting that these peptides have the potential to enhance Janus kinase (JAK)‐STAT signaling. In accordance with this finding, Pro‐Hyp has been shown to induce the phosphorylation of STAT3 in human dermal fibroblasts 45. Further studies are required to identify the receptors or target molecules for the peptides and elucidate the mechanism by which the peptides potentiate these intracellular signal transductions.

It has been demonstrated that collagen‐derived dipeptides and tripeptides are also generated endogenously at the sites of tissue damage and inflammation through degradation of collagen fibers 46. These oligopeptides are thought to act as so‐called danger signals, recruiting immune cells and fibroblasts to the site of inflammation and tissue injury. In inflammatory bowel disease and lung disease, proline‐glycine‐proline is produced by the combined action of matrix metalloproteinase and prolyl endoproteinase and exerts chemotactic activity for neutrophils 47, 48. Pro‐Hyp is generated in allergic contact dermatitis and can recruit and enhance the proliferation of skin fibroblasts, which contribute to wound healing 20. The present study indicated that collagen peptides generated at inflammation sites may control immune responses by modulating the balance of CD4^+^ T cells.

It has been reported that ingesting collagen peptides for eight weeks improved the immunological status of healthy Japanese men and women who had experienced daily tiredness and fatigue and improved their T‐cell parameters, including the number of T cells, memory T cells, CD8^+^CD28^+^ T cells, and the CD4/CD8 T‐cell ratio, suggesting that T‐cell function and development were modulated by collagen‐peptide ingestion 29. The present study indicated that ingestion of collagen peptides by mice skewed the balance of CD4^+^ T cells toward Th1 and Treg cells. Taken together, these studies suggest that ingesting collagen peptides modulates T‐cell function and development in vivo, improves immunological status, and/or suppresses allergic responses. However, further studies are required to elucidate the precise mechanisms of the effects of collagen‐peptide ingestion on immune functions.

In our in vitro experiments, the effects of Pro‐Hyp and Hyp‐Gly on skewing the CD4^+^ T‐cell balance toward Th1 and Treg cells were achieved at doses of 100–200 μM. This dose is physiologically relevant because approximately 100–140 μM of Hyp‐containing peptides have been reported as being detected in human plasma 2 h after ingestion of collagen peptide 12, 49. Furthermore, the U.S. Food and Drug Administration and other public organizations have recognized gelatin hydrolysate as safe 50. Overall, the present study suggested that collagen peptides seem to be a promising agent having both anti‐allergic and anti‐inflammatory actions in humans.

## Conflict of Interest

This study was supported by Nippi Inc. Yoh‐ichi Koyama, Chisa Tometsuka, Masashi Kusubata, Kumiko Kuwaba, Osamu Hayashida, and Shunji Hattori are employees of Nippi Inc.

## Supporting information

Additional supporting information may be found in the online version of this article at the publisher's web‐site.

**Figure S1**. Collagen peptide feeding did not affect mouse growth or water intake. Growth curve (A) and daily water intake (B) of mice fed with control diet and diet containing collagen peptide (n = 8). Values are means ± SEM.
**Figure S2**. Expression of peptide transporters in naive T cells. cDNA from naive BALB/c CD4^+^ T cells and fetus was amplified with PCR using specific primers for Pept1, Pept2, Pht1, and Ci1. Representative result of three independent experiments was shown.
**Figure S3**. Function of CD4^+^ CD25^+^ Treg cells differentiated in the presence of Pro‐Hyp peptide. (A) CD4^+^ CD25^+^ Treg cells differentiated with or without 200 μM Pro‐Hyp (PO) were incubated with CFSE labeled CD4^+^ T cells in the presence of anti‐CD3 and anti‐CD28. CFSEdilution was monitored by FACS 48 h after activation. The percentage of CFSE‐diluted are shown in the histograms. Quantitation data were shown in the graph (n = 3). (B) CD4^+^ CD25^+^ Treg cells differentiated with or without 200 μM Pro‐Hyp (PO) were stimulated with plate‐bound anti‐CD3 and anti‐CD28 for 3 days. IL‐10 produced in the culture supernatant was measured (n = 3). Values are means ± SEM.

## References

[1] Kay, A. B. 2001 Allergy and allergic diseases. N. Engl. J. Med. 344:30–37. 11136958 10.1056/NEJM200101043440106

[2] Romagnani, S. 2006 Regulation of the T cell response. Clin. Exp. Allergy 36:1357–1366. 17083345 10.1111/j.1365-2222.2006.02606.x

[3] Hamid, Q. , and M. Tulic . 2009 Immunobiology of asthma. Annu. Rev. Physiol. 71:489–507. 19575684 10.1146/annurev.physiol.010908.163200

[4] Murphy, K. M. , and S. L. Reiner . 2002 The lineage decisions of helper T cells. Nat. Rev. Immunol. 2:933–944. 12461566 10.1038/nri954

[5] O'Shea, J. J. , and W. E. Paul . 2010 Mechanisms underlying lineage commitment and plasticity of helper CD4^+^ T cells. Science. 327:1098–1102. 20185720 10.1126/science.1178334 PMC2997673

[6] Iwamoto, I. , H. Nakajima , H. Endo , and S. Yoshida . 1993 Interferon g regulates antigen‐induced eosinophil recruitment into the mouse airways by inhibiting the infiltration of CD4^+^ T cells. J. Exp. Med. 177:573–576. 8093895 10.1084/jem.177.2.573 PMC2190892

[7] Robinson, D. S. 2009 Regulatory T cells and asthma. Clin. Exp. Allergy. 39:1314–1323. 19538496 10.1111/j.1365-2222.2009.03301.x

[8] Palomares, O. , G. Yaman , A. K. Azkur , T. Akkoc , M. Akdis , and C. A. Akdis . 2010 Role of Treg in immune regulation of allergic diseases. Eur J Immnol. 40:1232–1240. 10.1002/eji.200940045 20148422

[9] Zague, V. 2008 A new view concerning the effects of collagen hydrolysate intake on skin properties. Arch Darmatol Res. 300:479–483. 10.1007/s00403-008-0888-4 18784933

[10] Moskowitz, R. W. 2000 Role of collagen hydrolysate in bone and joint disease. Semin. Arthritis Rheum. 30:87–99. 11071580 10.1053/sarh.2000.9622

[11] Ohara, H. , H. Matsumoto , K. Ito , K. Iwai , and K. Sato . 2007 Comparison of quantity and structures of hydroxyproline‐containing peptides in human blood after oral ingestion of gelatin hydrolysates from different sources. J. Agric. Food Chem. 55:1532–1535. 17253720 10.1021/jf062834s

[12] Iwai, K. , T. Hasegawa , Y. Taguchi , F. Morimatsu , K. Sato , Y. Nakamura , A. Higashi , Y. Kido , Y. Nakabo , and K. Ohtsuki . 2005 Identification of food‐derived collagen peptides in human blood after oral ingestion of gelatin hydrolysates. J. Agric. Food Chem. 53:6531–6536. 16076145 10.1021/jf050206p

[13] Ichikawa, S. , M. Morifuji , H. Ohara , H. Matsumoto , Y. Takeuchi , and K. Sato . 2010 Hydroxyproline‐containing dipeptides and tripeptides quantified at high concentration in human blood after oral administration of gelatin hydrolysate. Int. J. Food Sci. Nutr. 61:52–60. 19961355 10.3109/09637480903257711

[14] Shigemura, Y. , K. Iwai , F. Morimatsu , T. Iwamoto , T. Mori , C. Oda , T. Taira , E. Y. Park , Y. Nakamura , and K. Sato . 2009 Effect of Prolyl‐hydroxyproline (Pro‐Hyp), a food‐derived collagen peptide in human blood, on growth of fibroblasts from mouse skin. J. Agric. Food Chem. 57:444–449. 19128041 10.1021/jf802785h

[15] Ohara, H. , H. Iida , K. Ito , Y. Takeuchi , and Y. Nomura . 2010 Effects of Pro‐Hyp, a collagen hydrolysate‐derived peptide, on hyaluronic acid synthesis using in vitro cultured synovium cells and oral ingestion of collagen hydrolysates in a guinea pig model of osteoarthritis. Biosci. Biotechnol. Biochem. 74:2096–2099. 20944430 10.1271/bbb.100193

[16] Kimura, Y. , K. Ogura , Y. Taniuchi , A. Kataoka , N. Inoue , F. Sugihara , S. Nakatani , J. Shimizu , M. Wada , and H. Mano . 2014 Collagen‐derived dipeptide prolyl‐hydroxyproline promotes differentiation of MC3T3‐E1 osteoblastic cells. Biochem. Biophys. Res. Commun. 453:498–501. 25285626 10.1016/j.bbrc.2014.09.121

[17] Nakatani, S. , H. Mano , C. Sampei , J. Shimizu , and M. Wada . 2009 Chondroprotective effect of the bioactive peptide prolyl‐hydroxyproline in mouse articular cartilage in vitro and in vivo. Osteoarthritis Cartilage. 17:1620–1627. 19615963 10.1016/j.joca.2009.07.001

[18] Shigemura, Y. , S. Akaba , E. Kawashima , E. Y. Park , Y. Nakamura , and K. Sato . 2011 Identification of a novel food‐derived collagen peptide, hydroxyprolyl‐glycine, in human peripheral blood by pre‐column derivatisation with phenyl isothiocyanate. Food Chem. 129:1019–1024. 25212331 10.1016/j.foodchem.2011.05.066

[19] Kitakaze, T. , T. Sakamoto , T. Kitano , N. Inoue , F. Sugihara , N. Harada , and R. Yamaji . 2016 The collagen derived dipeptide hydroxyprolyl‐glycine promotes C2C12 myoblast differentiation and myotube hypertrophy. Biochem. Biophys. Res. Commun. 478:1292–1297. 27553280 10.1016/j.bbrc.2016.08.114

[20] Kusubata, M. , Y. Koyama , C. Tometsuka , Y. Shigemura , and K. Sato . 2015 Detection of endogenous and food‐derived collagen dipeptide prolylhydroxyproline (Pro‐Hyp) in allergic contact dermatitis‐affected mouse ear. Biosci. Biotechnol. Biochem. 79:1356–1361. 25848885 10.1080/09168451.2015.1027653

[21] Postlethwaite, A. E. , J. M. Seyer , and A. H. Kang . 1978 Chemotactic attraction of human fibroblasts to type I, II, and III collagens and collagen‐derived peptides. Proc. Natl. Acad. Sci. U. S. A. 75:871–875. 204938 10.1073/pnas.75.2.871 PMC411359

[22] Laskin, D. L. , T. Kimura , S. Sakakibara , D. J. Riley , and R. A. Berg . 1986 Chemotactic activity of collagen‐like polypeptides for human peripheral blood neutrophils. J. Luekoc. Biol. 39:255–266. 10.1002/jlb.39.3.255 3456007

[23] Postlethwaite, A. E. , and A. Kang . 1976 Collagen and collagen peptide‐induced chemotaxis of human blood monocytes. J. Exp. Med. 143:1299–1307. 1271012 10.1084/jem.143.6.1299 PMC2190221

[24] Tanaka, M. , Y. Koyama , and Y. Nomura . 2009 Effects of collagen peptide ingestion on UV‐B induced skin damage. Biosci. Biotechnol. Biochem. 73:930–932. 19352014 10.1271/bbb.80649

[25] Koyama, Y. , K. Kuwaba , S. Kondo , and Y. Tsukada . 2014 Supplemental ingestion of collagen peptide suppresses ultraviolet‐induced erythema. Jpn. Pharmacol. Ther. 42:781–789.

[26] Zhang, Z. , J. Wang , Y. Ding , X. Dai , and Y. Li . 2011 Oral administration of marine collagen peptides from Chum Salmon skin enhances cutaneous wound healing and angiogenesis in rats. J. Sci. Food Agric. 91:2173–2179. 21560132 10.1002/jsfa.4435

[27] Nakao, K. , M. Kusubata , K. Hara , M. Igarashi , N. Yamazaki , and Y. Koyama . 2013 Effects of collagen peptide ingestion on healing of skin wound in a rat model of pressure ulcer. Jpn. Pharmacol. Ther. 41:587–596.

[28] Shimizu, J. , N. Asami , A. Kataoka , F. Sugihara , N. Inoue , Y. Kimira , M. Wada , and H. Mano . 2015 Oral collagen‐derived dipeptides, prolyl‐hydroxyproline and hydroxyprolyl‐glycine, ameliorate skin barrier dysfunction and alter gene expression profiles in the skin. Biochem. Biophys. Res. Commun. 456:626–630. 25498544 10.1016/j.bbrc.2014.12.006

[29] Koyama, Y. , K. Kuwaba , M. Kusubata , O. Hayashida , T. Takara , and Y. Tsukada . 2015 Supplemental ingestion of collagen peptide improves T‐cell‐related human immune status. Jpn. Pharmacol. Ther. 43:51–56.

[30] Taketomi, Y. , N. Ueno , T. Kojima , H. Sato , R. Murase , K. Yamamoto , S. Tanaka , M. Sakanaka , M. Nakamura , Y. Nishito , et al. 2013 Mast cell maturation is driven via a group III phospholipase A2‐prostaglandin D2‐DP1 receptor paracrine axis. Nat. Immunol. 14:554–563. 23624557 10.1038/ni.2586 PMC4065307

[31] Tanaka, Y. , S. Hamano , K. Gotoh , Y. Murata , Y. Kunisaki , A. Nishikimi , R. Takii , M. Kawaguchi , A. Inayoshi , S. Masuko , et al. 2007 T helper type 2 differentiation and intracellular trafficking of the interleukin 4 receptor‐alpha subunit controlled by the Rac activator Dock2. Nat. Immunol. 8:1067–1075. 17767160 10.1038/ni1506

[32] Shibuya, S. , Y. Ozawa , T. Toda , K. Watanabe , C. Tometsuka , T. Ogura , Y. Koyama , and T. Shimizu . 2014 Collagen peptide and vitamin C additively attenuate age‐related skin atrophy in Sod1‐deficient mice. Biosci. Biotechnol. Biochem. 78:1212–1220. 25229861 10.1080/09168451.2014.915728

[33] Sun, C. M. , J. A. Hall , R. B. Blank , N. Bouladoux , M. Oukka , J. R. Mora , and Y. Belkaid . 2007 Small intestine lamina propria dendritic cells promote de novo generation of Foxp3 Treg cells via retinoic acid. J. Exp. Med. 204:1775–1785. 17620362 10.1084/jem.20070602 PMC2118682

[34] Barnes, M. J. , and F. Powrie . 2009 Regulatory T cells reinforce intestinal homeostasis. Immunity. 31:401–411. 19766083 10.1016/j.immuni.2009.08.011

[35] Rubtsov, Y. P. , J. P. Rasmussen , E. Y. Chi , J. Fontenot , L. Castelli , X. Ye , P. Treuting , L. Siewe , A. Roers , W. R. Henderson, Jr. , et al.. 2008 Regulatory T cell‐derived interleukin‐10 limits inflammation at environmental interfaces. Immunity. 28:546–558. 18387831 10.1016/j.immuni.2008.02.017

[36] Akdis, C. A. , T. Blesken , M. Akdis , B. Wüthrich , and K. Blaser . 1998 Role of interleukin 10 in specific immunotherapy. J. Clin. Invest. 102:98–106. 9649562 10.1172/JCI2250 PMC509070

[37] Thompson‐Snipes, L. , V. Dhar , M. W. Bond , T. R. Mosmann , K. W. Moore , and D. M. Rennick . 1991 Interleukin 10: a novel stimulatory factor for mast cells and their progenitors. J. Exp. Med. 173:507–510. 1899106 10.1084/jem.173.2.507 PMC2118779

[38] Schandene, L. , C. Alonso‐Vega , F. Willems , C. Gerard , A. Delvaux , T. Velu , R. Devos , M. de Boer , and M. Goldman . 1994 B7/CD28‐dependent IL‐5 production by human resting T cells is inhibited by IL‐10. J. Immunol. 152:4368–4374. 7512591

[39] Kawaguchi, T. , P. N. Nanbu , and M. Kurokawa . 2012 Distribution of prolylhydroxyproline and its metabolites after oral administration in rats. Biol. Pharm. Bull. 35:422–427. 22382331 10.1248/bpb.35.422

[40] Gajewski, T. F. , and F. W. Fitch . 1998 Anti‐proliferative effect of IFN‐g in immune regulation. I. IFN‐g inhibits the proliferation of Th2 but not Th1 murine helper T lymphocyte clones. J. Immunol. 140:4245–4252. 2967332

[41] Mitchell, C. , K. Provost , N. Niu , R. Homer , and L. Cohn . 2011 IFN‐g acts on the airway epithelium to inhibit local and systemic pathology in allergic airway disease. J. Immunol. 187:3815–3820. 21873527 10.4049/jimmunol.1100436 PMC3178669

[42] Prigione, I. , F. Morandi , M. A. Tosca , M. Silvestri , V. Pistoia , G. Ciprandi , and G. A. Rossi . 2010 Interferon‐gamma and IL‐10 may protect from allergic polysensitization in children: preliminary evidence. Allergy. 65:740–742. 19958312 10.1111/j.1398-9995.2009.02285.x

[43] Watanabe‐Kamiyama, M. , M. Shimizu , S. Kamiyama , Y. Taguchi , H. Sone , F. Morimatsu , H. Shirakawa , Y. Furukawa , and M. Komai . 2010 Absorption and effectiveness of orally administered low molecular weight collagen hydrolysate in rats. J. Agric. Food Chem. 58:835–841. 19957932 10.1021/jf9031487

[44] Smith, D. E. , B. Clémençon , and M. A. Hediger . 2013 Proton‐coupled oligopeptide transporter family SLC15: Physiological, pharmacological and pathological implications. Mol. Aspects Med. 34:323–336. 23506874 10.1016/j.mam.2012.11.003 PMC3602806

[45] Ohara, H. , S. Ichikawa , H. Matsumoto , M. Akiyama , N. Fujimoto , T. Kobayashi , and S. Tajima . 2010 Collagen‐derived dipeptide, proline‐hydroxyproline, stimulates cell proliferation and hyaluronic acid synthesis in cultured human dermal fibroblasts. J. Dermatol. 37:330–338. 20507402 10.1111/j.1346-8138.2010.00827.x

[46] Koelink, P. J. , S. A. Overbeek , S. Braber , M. E. Morgan , P. A. Henricks , M. Abdul Roda , H. W. Verspaget , S. C. Wolfkamp , A. A. te Velde , C. W. Jones , et al. 2014 Collagen degradation and neutrophilic infiltration: a vicious circle in inflammatory bowel disease. Gut. 63:578–587. 23525573 10.1136/gutjnl-2012-303252 PMC3963538

[47] Weathington, N. M. , A. H. van Houwelingen , B. D. Noerager , P. L. Jackson , A. D. Kraneveld , F. S. Galin , G. Folkerts , F. P. Nijkamp , and J. E. Blalock . 2006 A novel peptide CXCR ligand derived from extracellular matrix degradation during airway inflammation. Nat. Med. 12:317–323. 16474398 10.1038/nm1361

[48] Gaggar, A. , P. L. Jackson , B. D. Noerager , P. J. O'Reilly , D. B. McQuaid , S. M. Rowe , J. P. Clancy , and J. E. Blalock . 2008 A novel proteolytic cascade generates an extracellular matrix‐derived chemoattractant in chronic neutrophilic inflammation. J. Immunol. 180:5662–5669. 18390751 10.4049/jimmunol.180.8.5662 PMC3998665

[49] Taga, Y. , M. Kusubata , K. Ogawa‐Goto , and S. Hattori . 2014 Highly accurate quantification of hydroxyproline‐containing peptides in blood using a protease digest of stable isotope‐labeled collagen. J. Agric. Food Chem. 62:12096–12102. 25417748 10.1021/jf5039597

[50] Sibilla, S. , M. Godfrey , S. Brewer , A. Budh‐Raja , and L. Genovese . 2015 An overview of the beneficial effects of hydrolysed collagen as a nutraceutical on skin properties: scientific background and clinical studies. Open Nutraceuticals J. 8:29–42.

