# Supplementary material for: Collagen‐derived peptides modulate CD4+ T‐cell differentiation and suppress allergic responses in mice
Source: Immun Inflamm Dis. 2018 Feb 1;6(2):245–55. doi: 10.1002/iid3.213 (PMC5946155; doi:10.1002/iid3.213)
Supplement: Supplementary file 1 — Figure S1. Collagen peptide feeding did not affect mouse growth or water intake. Growth curve (A) and daily water intake (B) of mice fed with control diet and diet containing collagen peptide (n = 8). Values are means ± SEM. Figure S2. Expression of peptide transporters in naive T cells. cDNA from naive BALB/c CD4+ T cells and fetus was amplified with PCR using specific primers for Pept1, Pept2, Pht1, and Ci1. Representative result of three independent experiments was shown. Figure S3. Function of CD4+ CD25+ Treg cells differentiated in the presence of Pro‐Hyp peptide. (A) CD4+ CD25+ Treg cells differentiated with or without 200 μM Pro‐Hyp (PO) were incubated with CFSE labeled CD4+ T cells in the presence of anti‐CD3 and anti‐CD28. CFSEdilution was monitored by FACS 48 h after activation. The percentage of CFSE‐diluted are shown in the histograms. Quantitation data were shown in the graph (n = 3). (B) CD4+ CD25+ Treg cells differentiated with or without 200 μM Pro‐Hyp (PO) were stimulated with plate‐bound anti‐CD3 and anti‐CD28 for 3 days. IL‐10 produced in the culture supernatant was measured (n = 3). Values are means ± SEM. [file IID3-6-245-s001.pdf]

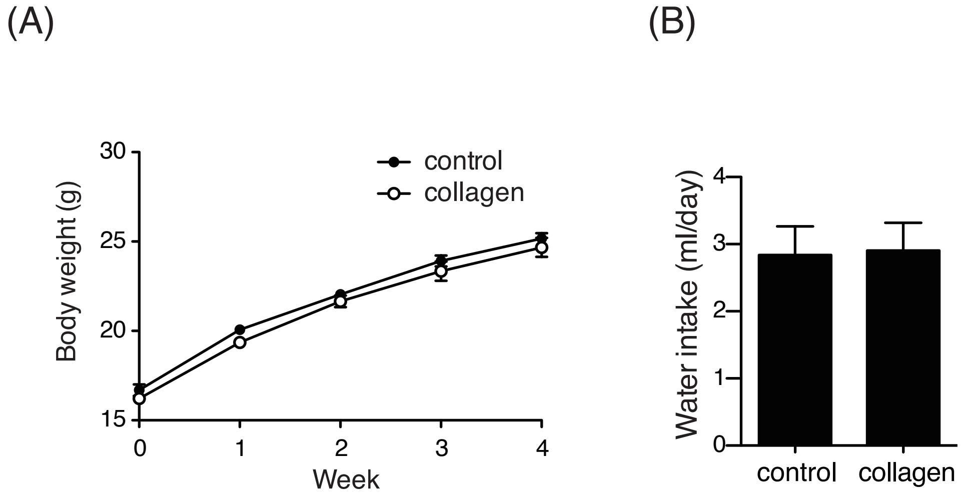

Figure S1. Collagen peptide feeding did not affect mouse growth or water intake. Growth curve (A) and daily water intake (B) of mice fed with control diet and diet containing collagen peptide (n = 8). Values are means ± SEM.

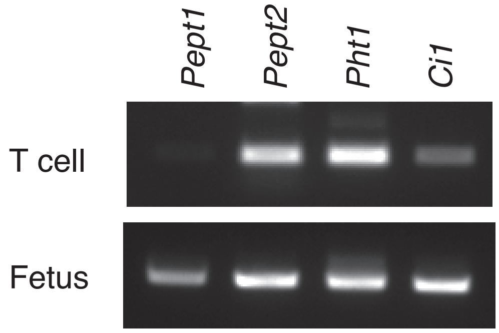

Figure S2. Expression of peptide transporters in naive T cells. cDNA from naive BALB/c CD4$^+$ T cells and fetus was amplified with PCR using specific primers for *Pept1*, *Pept2*, *Pht1*, and *Ci1*. Representative result of three independent experiments was shown.

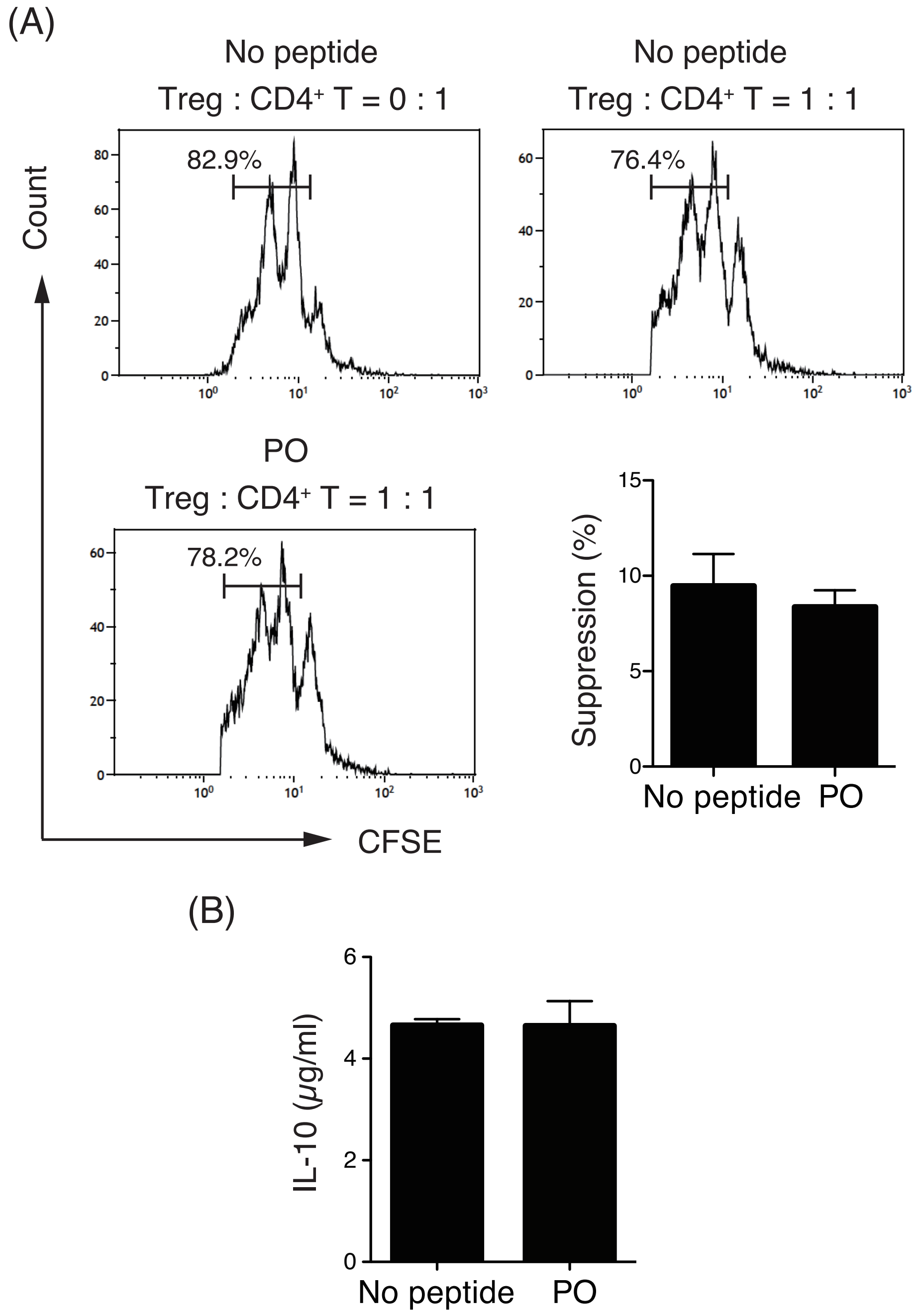

Figure S3. Function of $CD4^+$ $CD25^+$ Treg cells differentiated in the presence of Pro-Hyp peptide. (A) $CD4^+$ $CD25^+$ Treg cells differentiated with or without 200 μM Pro-Hyp (PO) were incubated with CFSE labeled $CD4^+$ T cells in the presence of anti-CD3 and anti-CD28. CFSE dilution was monitored by FACS 48 h after activation. The percentage of CFSE-diluted are shown in the histograms. Quantitation data were shown in the graph (n = 3). (B) $CD4^+$ $CD25^+$ Treg cells differentiated with or without 200 μM Pro-Hyp (PO) were stimulated with plate-bound anti-CD3 and anti-CD28 for 3 days. IL-10 produced in the culture supernatant was measured (n = 3). Values are means ± SEM.
